# Clinical Significance of Somatic *PIK3CA* and *MAP3K3* Mutations in Cerebral and Spinal Cavernous Malformations

**DOI:** 10.1007/s12975-025-01360-2

**Published:** 2025-06-06

**Authors:** Hiroki Hongo, Satoru Miyawaki, Keisuke Takai, Hideaki Ono, Masahiro Shimizu, Takashi Matsukawa, Shotaro Ogawa, Yu Teranishi, Satoshi Kiyofuji, Kenta Ohara, Daiichiro Ishigami, Yu Sakai, Seiei Torazawa, Yudai Hirano, Daisuke Shimada, Naoto Kunii, Seijiro Shimada, Jun Mitsui, Hiroto Katoh, Daisuke Komura, Hirofumi Nakatomi, Shumpei Ishikawa, Nobuhito Saito

**Affiliations:** 1 Department of Neurosurgery, Faculty of Medicine, The University of Tokyo, Tokyo, Japan; 2 Department of Neurosurgery, Tokyo Metropolitan Neurological Hospital, Fuchu, Japan; 3 Department of Neurosurgery, Fuji Brain Institute and Hospital, Fujinomiya, Japan; 4 Department of Neurosurgery, Kanto Neurosurgical Hospital, Kumagaya, Japan; 5 Department of Neurology, Graduate School of Medicine, The University of Tokyo, Tokyo, Japan; 6 Department of Neurosurgery, Kyorin University Faculty of Medicine, Mitaka, Japan; 7 Department of Neurosurgery, Jichi Medical University, Shimotsuke, Japan; 8 Department of Precision Medicine Neurology, Graduate School of Medicine, The University of Tokyo, Tokyo, Japan; 9 Department of Preventive Medicine, Graduate School of Medicine, The University of Tokyo, Tokyo, Japan

**Keywords:** Cavernous malformations, Hemorrhage, *MAP3K3*, *PIK3CA*, Somatic mutations

## Abstract

Somatic *PIK3CA* and *MAP3K3* mutations in cerebral and spinal cavernous malformations (CMs) have been identified in recent studies. However, their significance in the clinical presentation and risk of hemorrhage in CMs remains poorly understood. We aimed to analyze the association between these mutations and the clinical characteristics of CMs. Among patients with CMs who underwent surgical resection of lesions between July 2002 and March 2022, those with complete clinical and radiological data at the time of initial surgery were included. Somatic *PIK3CA* and *MAP3K3* mutations were detected using droplet digital polymerase chain reaction. Subsequently, the clinical and radiological characteristics correlated with these mutations were assessed. Furthermore, the effect of these mutations on the first symptomatic intraparenchymal hemorrhage during follow-up was evaluated. In total, 72 patients were included; among them, 50 had sufficient mutation data. *PIK3CA* E542K, E545K, and H1047R mutations were identified in 7 (14%), 7 (14%), and 15 (30%) patients, respectively. *MAP3K3 *I441M was identified in 10 (20%) patients (8 [16%] had both *PIK3CA* and *MAP3K3* mutations). *MAP3K3 *I441M was more common in patients with Zabramski classification type II lesions than in those with CMs of other types (*p* = 0.024). Multivariate Cox regression analyses identified the presence of a *PIK3CA* mutation as a risk factor for early (re)hemorrhage. The results suggest that *PIK3CA* and *MAP3K3* mutations are associated with clinical and radiological characteristics in patients with CMs and that the presence of a somatic *PIK3CA* mutation increases susceptibility to hemorrhage. These findings may help guide future therapeutic strategies.

**Supplementary Information:**

The online version contains supplementary material available at 10.1007/s12975-025-01360-2.

## Introduction

Cerebral cavernous malformations (CCMs) and spinal cord cavernous malformations (SCCMs) are vascular anomalies of the central nervous system that are composed of abnormally dilated thin-walled capillaries and veins. They are the second most common type of neurovascular malformation, a prevalence of 0.16–0.40% in the general population [1]. Cavernous malformations (CMs) present in either familial (approximately 20% of cases) or sporadic (approximately 80% of cases) forms [2]. Familial CMs are caused by biallelic, inherited germline and additional somatic, loss-of-function mutations in one of three genes: *CCM1* (*KRIT1*), *CCM2*, and *CCM3* (*PDCD10*) [3]. Basic molecular studies have revealed that these proteins play roles in proper vascular development by negatively regulating the MEKK3-KLF2/4 (downstream transcription factor effectors of MEKK3) signaling pathway in vascular endothelial cells [4]. In addition to these mutations, somatic *PIK3CA* and *MAP3K3* mutations in CM lesions have been identified in recent studies [1, 5–7]. Whereas familial CMs are primarily caused by mutations in one of the *CCM1*, *CCM2*, or *CCM3* genes, sporadic CMs, which account for the majority of cases, more commonly harbor somatic mutations in *PIK3CA* and *MAP3K3* [5, 8]. Studies conducted using mouse models have demonstrated that these somatic mutations contribute to the formation of CMs [6, 9, 10].

CMs are a major source of intracerebral/intramedullary hemorrhage that can cause serious neurological sequelae. Notably, the bleeding rate of CMs varies widely among patients [11]. A history of hemorrhage increases the likelihood of a subsequent hemorrhage [11, 12]. Moreover, studies have reported that the location in the brainstem significantly affects susceptibility to hemorrhage [11, 12]. In addition, some studies have demonstrated that SCCMs are more susceptible to hemorrhage than CCMs and even brainstem CMs [13–16]. Other characteristics such as the female sex [17, 18], larger lesion size [19–21], and presence of associated developmental venous anomaly (DVA) [19, 21, 22] have also been reported to increase the risk for hemorrhage in patients with CMs. Furthermore, Zabramski class has also been indicated as a factor associated hemorrhage. The Zabramski classification was originally developed to categorize CCM lesions based on their appearance on magnetic resonance imaging (MRI), and it is currently the most widely used radiological classification system for CMs [23]. It classifies lesions according to their signal intensities on T1-weighted imaging (T1WI), T2-weighted imaging (T2WI), and gradient-echo sequences, as follows: type I, acute or subacute hemorrhage; type II, a typical 'mulberry' appearance with small hemorrhages and thromboses of varying ages; type III, chronic hemorrhage; and type IV, dot-sized lesions [24]. Several studies have reported that Zabramski type I (and type II) lesions are associated with an increased risk of future hemorrhage [23, 25, 26]. In contrast, dot-sized familial CMs and incidentally discovered lesions are usually quiescent [27, 28]. The findings from previous studies indicate that CM is highly heterogeneous, with different types of lesions displaying distinct characteristics. The clinical and radiological characteristics of CMs and their susceptibility to hemorrhage may be affected by their molecular features, including the presence of somatic *PIK3CA* and *MAP3K3* mutations, which were detected in recent studies. A recent study indicated that *PIK3CA* and *MAP3K3* mutations are more common in cerebral and spinal CMs, respectively [1]. *MAP3K3 *I441M*, a* hotspot mutation, is found exclusively in solitary lesions and predominantly in Zabramski type II and III lesions [7, 29, 30]. Several previous studies have indicated that lesions carrying these mutations may have different levels of susceptibility to hemorrhage. A few studies identified that lesions with *PIK3CA* mutations are more prone to bleeding than those with *MAP3K3 *I441M mutations, whereas another study demonstrated that lesions with *MAP3K3 *I441M are less prone to hemorrhage than lesions with CCM gene mutations [1, 8, 30]. However, the clinical significance of somatic *PIK3CA* and *MAP3K3* mutations has not been extensively investigated, despite their presumed role as major contributors to most cases of CMs, as they have been only recently identified. Therefore, this study aimed to evaluate the association between these mutations and the clinical and radiological characteristics of CMs.

## Methods

### Study Population

**Fig. 1:**
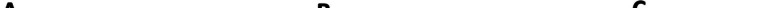
Typical appearance on MRI for each Zabramski classification type. **A** Type I lesion in the brainstem (Patient 16 at the time of surgery), defined as a unilocular lesion that appears hyperintense on T1-weighted imaging (T1WI) and shows a hyperintense or hypointense core on T2-weighted imaging (T2WI), consistent with acute or subacute hemorrhage. **B** Type II lesion in the right frontal lobe (Patient 32 at the time of surgery), defined as a lesion exhibiting a reticulated signal core on both T1WI and T2WI, representing hemorrhage and thrombosis of varying ages. **C** Type III lesion in the left temporal lobe (white arrows; Patient 26 at the time of surgery), defined as a lesion that appears isointense on T1WI and hypointense on T2WI, consistent with chronic hemorrhage. T1WI images are shown on the left and T2WI images on the right. Type IV CM lesions were not identified in this study

This observational study included patients with CMs who underwent surgical lesion resection between July 2002 and March 2022 at the University of Tokyo Hospital (Tokyo, Japan), Tokyo Metropolitan Neurological Hospital (Fuchu, Japan), Fuji Brain Institute and Hospital (Fujinomiya, Japan), and Kanto Neurosurgical Hospital (Kumagaya, Japan), that fulfilled the following inclusion criteria: MRI data and clinical characteristics available at the time of surgery and provision of informed consent. Patients who had undergone surgery for recurrent lesions or had a history of prior brain irradiation were excluded, as these interventions may influence the native genomic status or alter the intrinsic behavior of lesions. CMs were diagnosed on the basis of the lesions’ typical appearance on MRI and pathological examination of the resected lesions. The following clinical and radiological data were extracted from the patients’ charts: age at the time of diagnosis and surgery, sex, number of lesions at the time of diagnosis and surgery, mode of presentation leading to diagnosis and surgery (hemorrhage, epileptic seizure, others), size of lesion at the time of diagnosis and surgery, MRI appearance at the time of diagnosis and surgery, location of resected lesions, and the presence of associated DVA. We defined hemorrhage as a symptomatic event with radiographic evidence of overt bleeding. The Zabramski classification was used to categorize the lesions into three types (type I, type II, and type III) based on their appearance on MRI, according to the original classification (Fig. 1) [24]. Type IV CM lesions were not identified in this study. The presence of DVA was based on MRI findings, including T2-weighted, susceptibility-weighted, and contrast-enhanced T1-weighted images. Two neurosurgeons independently reviewed the entire dataset to ensure accurate data acquisition.

### Mutation Analysis

Droplet digital polymerase chain reaction (ddPCR) was performed as previously described [31]. DNA was extracted from frozen or formalin-fixed paraffin-embedded (FFPE) CM tissue samples using commercially available DNA extraction kits (QIAamp DNA Micro Kit and QIAGEN QIAamp DNA FFPE Tissue Kit, respectively) (QIAGEN, Venlo, The Netherlands) following the manufacturer’s protocol. Detection of *PIK3CA* E542 K, E545 K, and H1047R, the most common *PIK3CA* mutations, and *MAP3K3 *I441M was performed using the QX200 Droplet Digital PCR system (Bio-Rad Laboratories, Inc., Hercules, CA, United States). Commercially available assay kits were used for the detection of *PIK3CA* mutations (Bio-Rad; dHsaMDV2010073, dHsaMDV2010075, and dHsaMDV2010077 for E542 K, E545 K, and H1047R, respectively). Customized primers and probes were used to detect *MAP3K3 *I441M (Bio-Rad; dHsaMDS106814991). The reaction mix consisted of 10 μL of ddPCR Supermix for Probes (no dUTP) (Bio-Rad), 1 μL of each ddPCR assay, genomic DNA (40 ng for frozen tissues or 40 ng or more for FFPE tissue to ensure sufficient copies of the target were obtained from fragmented DNA), and up to 20 μL of DNase/Rnase-free water. PCR was performed using a Bio-Rad S1000 Thermal Cycler. The cycling conditions for *PIK3CA* mutations were per the manufacturer’s recommended protocol. The cycling conditions for *MAP3K3 *I441M were 95 °C for 10 min, followed by 40 cycles of 94 °C for 30 s and 60 °C for 1 min, then 98 °C for 10 min, and finally a 4 °C hold. The PCR products were analysed using a QX200 droplet reader and QuantaSoft software (Bio-Rad). Each assay included wild-type (WT) and mutation-positive controls for each mutation, synthesized Block Gene Fragment (Integrated DNA Technologies, Coralville, IA, United States), and a no-template control. Results of ddPCR of samples that did not have ≥ 500 droplets for each WT were considered unreliable and excluded from the analysis. A sample was considered positive if it had a minimum of 1% fractional abundance. For the detection of *PIK3CA* E542 K and E545 K, FFPE DNA samples were treated with uracil DNA glycosylase (New England Biolabs, Ipswich, MA, United States) before PCR to reduce the risk of false positives, as previously described [32]. This treatment is necessary because formalin fixation can introduce false positives for these mutations (G > A substitutions).

### Statistical Analysis

Continuous variables are expressed as medians and ranges, and categorical variables are expressed as percentages. For continuous variables, the unpaired or paired Wilcoxon rank-sum test was used for pairwise comparisons, whereas the Kruskal–Wallis test was used for comparisons of three groups. Categorical variables were compared using the chi-square test or Fisher’s exact test, as appropriate. Kaplan–Meier survival analysis was performed using the log-rank test. Cox regression analyses were used to assess the time-to-event of the first hemorrhage since the time of diagnosis. In the Cox regression analyses, univariate analysis of all variables was performed, and the identified confounders were included in the multivariate analysis. The date of diagnosis was used as the starting point, and data were censored on the date of surgery. JMP Pro ver. 18 was used for all statistical analyses. Statistical significance was set at *p* < 0.05.

## Results

### Patient Characteristics and Mutational Profiles

A total of 72 patients with CMs were included in this study. Among them, two who underwent surgery for recurrence were excluded. Additionally, 20 patients whose samples were of inadequate quality, which resulted in an insufficient number of droplets for WT in ddPCR analyses, were excluded. Fifty patients with complete clinical and radiological information at the time of surgery and ddPCR data were eligible for analysis. Of these, 43 had cerebral CMs and 7 had spinal cord CMs (figures, Online Resources 1–3). Among the patients’ samples, 37 were frozen and 13 were FFPE samples. *PIK3CA* mutations were identified in 29 (58%) samples. The E542 K, E545 K, and H1047R mutations were detected in 7 (14%), 7 (14%), and 15 (30%) samples, respectively. *MAP3K3 *I441M was identified in 10 (20%) samples. Of these, eight (16%) samples had both one *PIK3CA* mutation and *MAP3K3 *I441M (figures, Online Resource 4). We classified the patients into four groups based on the mutations identified in their samples: *PIK3CA*-mutation-positive, *MAP3K3 *I441M-positive, double-positive, and double-negative. The clinical and radiological characteristics of the patients were compared according to these groups (Table 1). We found that the age at the time of surgery and the frequency of associated DVA were significantly different among the groups (*p* = 0.045 and 0.022, respectively).

**Table 1:** Clinical and radiological characteristics of patients at the time of surgery (n = 50) stratified according to their somatic mutation status

Characteristics	Overall	*PIK3CA-*mutation-positive	*MAP3K3 *I441M-positive	Double positive	Double negative	*p* value
Number of patients	50	21	2	8	19	-
Age,^a^ years (median, range)	42, 12–79	35, 12–73	24.5, 24–25	47.5, 22–69	46, 15–79	**0.045**
Sex, n (%)						0.200
Female	36 (72.0%)	18 (85.7%)	2 (100%)	5 (62.5%)	11 (57.9%)	
Male	14 (28.0%)	3 (14.3%)	0 (0%)	3 (37.5%)	8 (42.1%)	
Number of lesions,^a^ n (%)						0.421
Single	40 (80.0%)	16 (76.2%)	2 (100%)	8 (100%)	14 (73.7%)	
Multiple	10 (20.0%)	5 (23.8%)	0 (0%)	0 (0%)	5 (26.3%)	
Mode of presentation,^a^ n (%)						0.726
Hemorrhage	39 (78.0%)	18 (85.7%)	2 (100%)	6 (75.0%)	13 (68.4%)	
Epileptic seizure	9 (18.0%)	2 (9.5%)	0 (0%)	2 (25.0%)	5 (26.3%)	
Lesion enlargement	2 (4%)	1 (4.8%)	0 (0%)	0 (0%)	1 (5.3%)	
Size,^a^ mm (median, range)	22.5, 3–46	25, 8–46	19.5, 6–33	14.5, 5–28	21, 3–37	*0.095*
Zabramski classification,^a^ n (%)						0.100
Type I	26 (52.0%)	12 (57.1%)	1 (50%)	1 (12.5%)	12 (63.2%)	
Type II	23 (46.0%)	8 (38.1%)	1 (50%)	7 (87.5%)	7 (36.8%)	
Type III	1 (2.0%)	1 (4.8%)	0 (0%)	0 (0%)	0 (0%)	
Type IV	0 (0%)	0 (0%)	0 (0%)	0 (0%)	0 (0%)	
Lesion location, n (%)						0.108
Non-brainstem, intracranial	22 (44.0%)	10 (47.6%)	0 (0%)	3 (37.5%)	9 (47.4%)	
Brainstem	21 (42.0%)	11 (52.3%)	1 (50%)	3 (37.5%)	6 (31.6%)	
Spinal cord	7 (14.0%)	0 (0%)	1 (50%)	2 (25.0%)	4 (21.0%)	
Associated DVA, n (%)						**0.022**
Present	21 (42.0%)	14 (66.7%)	0 (0%)	2 (25.0%)	5 (26.3%)	
Absent	29 (58.0%)	7 (33.3%)	2 (100%)	6 (75.0%)	14 (73.7%)	

^a^At the time of surgery*DVA* developmental venous anomaly

### Characteristics of Patients with *PIK3CA* Mutations or *MAP3K3* I441M: Analysis at the time of surgery

We compared the clinical and radiological factors between patients with and without *PIK3CA* or *MAP3K3* mutations, assessing the effect of each mutation independently, irrespective of the presence of the other (Tables 2 and 3). Associated DVA was more common in patients with *PIK3CA* mutations than in those without *PIK3CA* mutations (*p* = 0.027). The presence of the *MAP3K3 *I441M mutation was less common in Zabramski type I lesions than in lesions of other types (*p* = 0.024). All lesions with *MAP3K3 *I441M were obtained from patients with single lesions.

**Table 2:** Comparison of clinical and radiological characteristics between patients with and without *PIK3CA* mutations at the time of surgery (n = 50)

Characteristics	*PIK3CA* mutation		*p* value
	Positive	Negative	
Number of patients	29	21	
Age,^a^ continuous (median, range)	41, 12–73	42, 15–79	0.340
Sex, female, n (%)	23 (79.3%)	13 (61.9%)	0.176
Number of lesions,^a^ multiple, n (%)	5 (17.2%)	5 (23.8%)	0.567
Mode of presentation,^a^ hemorrhage, n (%)	24 (82.8%)	15 (71.4%)	0.636
Size,^a^ continuous (median, range)	23, 5–46	21, 3–37	0.522
Zabramski classification,^a^ type I, n (%)	13 (44.8%)	13 (61.9%)	0.233
Lesion location, brainstem and spinal cord, n (%)	15 (51.7%)	14 (66.7%)	0.890
Associated DVA, n (%)	16 (55.2%)	5 (23.8%)	**0.027**

^a^At the time of surgery *DVA,* developmental venous anomaly

**Table 3:** Comparison of clinical and radiological characteristics between patients with and without *MAP3K3 *I441M at the time of surgery (n = 50)

Characteristics	*MAP3K3* I441M		*p* value
	Positive	Negative	
Number of patients	10	40	
Age,^a^ continuous (median, range)	44.5, 22–69	41.5, 12–79	0.752
Sex, female, n (%)	7 (70.0%)	29 (72.5%)	1.00
Number of lesions,^a^ multiple, n (%)	0 (0%)	10 (25.0%)	0.179
Mode of presentation,^a^ hemorrhage, n (%)	8 (80.0%)	31 (77.5%)	1.00
Size,^a^ continuous (median, range)	14.5, 5–33	23.5, 3–46	*0.051*
Zabramski classification,^a^ type I, n (%)	2 (20%)	24 (60%)	**0.024**
Lesion location, brainstem and spinal cord, n (%)	6 (60.0%)	23 (57.5%)	0.480
Associated DVA, n (%)	2 (20.0%)	19 (47.5%)	0.160

^a^At the time of surgery *DVA,* developmental venous anomaly

### Mutation Allele Frequencies of *PIK3CA* and *MAP3K3* Mutations

We further investigated the mutation allele frequencies (MAFs) of *PIK3CA* and *MAP3K3* mutations identified in the patients. The MAFs of *PIK3CA* mutations and *MAP3K3 *I441M were 2.8% (1.0%–13.0%) and 4.0% (1.5%–9.7%), respectively. These values were not significantly different (figure, Online Resource 5 A). Analysis performed using the paired Wilcoxon rank-sum test showed that in patients with both *PIK3CA* and *MAP3K3* mutations, the MAFs of the two genes were not significantly different (figure, Online Resource 5B). We analyzed the association between MAF and clinical/radiological characteristics separately among patients with *PIK3CA* or *MAP3K3* mutations (figure, Online Resource 6). The results indicated that females with *MAP3K3 *I441M have significantly lower MAFs than males with the same mutation (*p* = 0.017). The same trend was observed for *PIK3CA* mutations (*p* = 0.056). The MAFs of *PIK3CA* mutations were significantly different between Zabramski classifications (*p* = 0.035); they were significantly lower in type I lesions than in type II lesions (*p* = 0.012).

### Analysis of Risk Factors for Hemorrhage During Follow-up

**Fig. 2:**
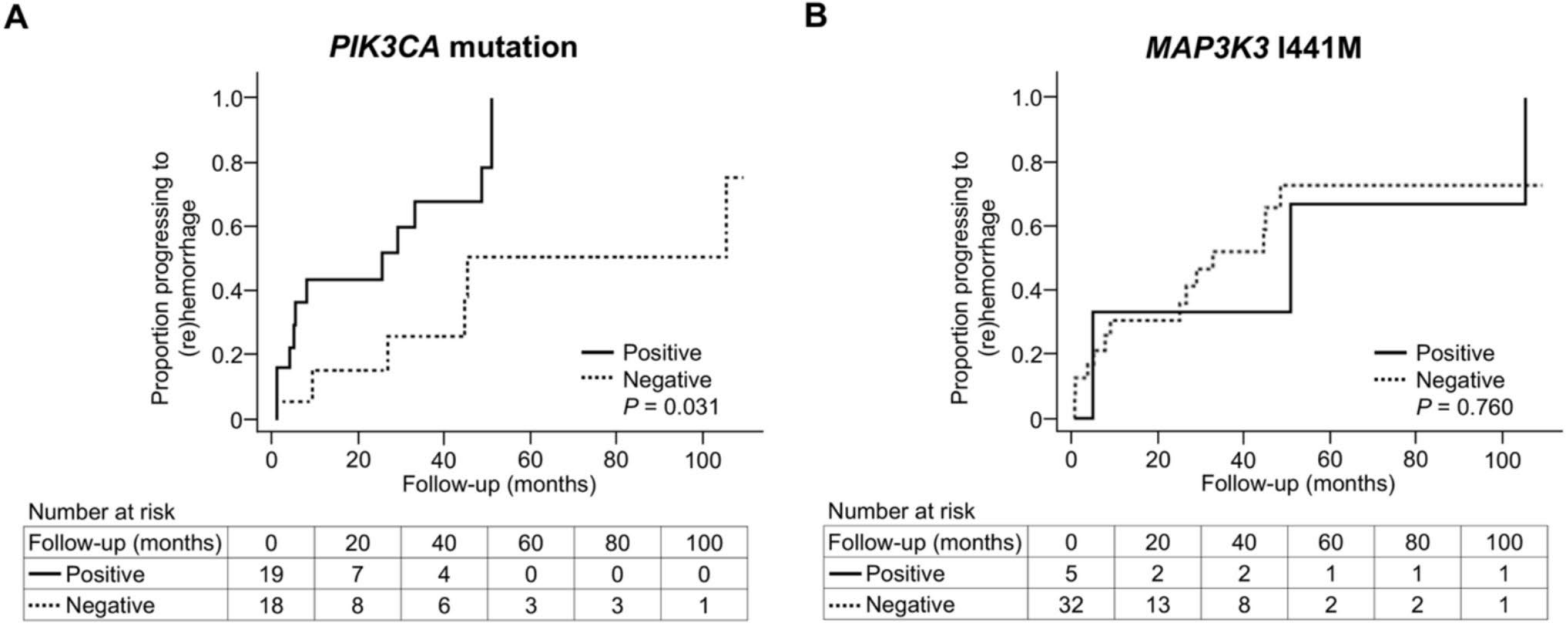
Kaplan–Meier plots of progression to intracerebral/spinal hemorrhage in the analysis of risk factors for hemorrhage during follow-up (n = 37). **A** Thirty-seven patients were divided into two groups based on the presence or absence of *PIK3CA* mutations. The"Positive"group consisted of patients with *PIK3CA* mutations, while the "Negative" group consisted of those without *PIK3CA* mutations. **B** Thirty-seven patients were divided into two groups based on the presence or absence of *MAP3K3 *I441M mutations. The "Positive" group consisted of patients with the *MAP3K3 *I441M mutation, and the "Negative" group consisted of those without it. Note: In both panels, the groups were defined solely based on the presence or absence of the specific mutation mentioned, regardless of the presence or absence of the other mutation. As a result, a patient could belong to the "Positive" or "Negative" group for each mutation independently. The horizontal axis represents follow-up time (months), and the vertical axis represents the proportion of patients progressing to hemorrhage after diagnosis. These plots illustrate the cumulative hemorrhage rate (1 − survival rate) over time. The “number at risk” at each time point indicates the number of individuals who have not yet experienced the event up to that point. In panel A, the difference first reached statistical significance at 33 months (P = 0.037). It temporarily lost significance at 45 months (P = 0.065) but became significant again at 51 months (P = 0.031) and remained so thereafter

We examined the association between *PIK3CA* and *MAP3K3* mutations and hemorrhage during the follow-up after CM diagnosis. Given that it is difficult to determine the presence or absence of *PIK3CA* and *MAP3K3* mutations in each lesion at the time of diagnosis, we considered lesions with these mutations at the time of surgery to have had the same mutations at the time of diagnosis. Thereafter, we analyzed the risk factors for hemorrhage during follow-up using mutational data and clinical and radiological information collated from the time of diagnosis. Of the 50 patients with available mutational data, 13 were excluded due to insufficient radiological information at the time of diagnosis, which made it impossible to accurately determine their clinical status and the timing of diagnosis. The remaining 37 patients were included in the analysis (figure, Online Resources 1,2 and 7–9). To assess the association between each clinical, radiological, and mutational factor and hemorrhage during follow-up, we divided patients into two groups for each factor and performed comparative analyses. The results showed that patients who had lesions with *PIK3CA* mutations developed symptomatic (re)hemorrhage significantly earlier than those without *PIK3CA* mutations (*p* = 0.031) (Fig. 2A, table, Online Resource 11). In contrast, no significant difference was noted between patients with *MAP3K3 *I441M and those without the mutation (Fig. 2B, table, Online Resource 11). Other than the presence of *PIK3CA* mutations, older age (≥ 35 years, median age at diagnosis) (*p* = 0.015), single lesions (*p* = 0.030), presentation of hemorrhage at diagnosis (*p* = 0.030), and lesions located in the brainstem or spinal cord (*p* = 0.011) were significantly associated with a shorter period between CM diagnosis and the first hemorrhage (figure and table, Online Resource 10 and 11). In addition, multivariate Cox regression analysis revealed that a *PIK3CA* mutation (hazard ratio: 2.96 [95% confidence interval: 1.03–8.49]; *p* = 0.044) was a significant risk factor for early hemorrhage after diagnosis (Table 4).

**Table 4:** Cox regression analysis of risk factors for hemorrhage during follow-up (n = 37)

Characteristics	Univariable		Multivariable	
	*p* value	Hazard ratio (95% CI)	*p* value	Hazard ratio (95% CI)
Age,^a^ ≥ 35 years	**0.021**	**3.56 (1.21–10.5)**	0.152	2.33 (0.73–7.37)
Sex, female	0.789	0.88 (0.34–2.28)	NA	NA
Number of lesions,^a^ single	0.061	6.97 (0.92–52.9)	NA	NA
Mode of presentation,^a^ hemorrhage	**0.042**	**3.65 (1.05–12.7)**	0.997	1.00 (0.15–6.74)
Size,^a^ ≥ 15 mm	0.560	0.74 (0.27–2.02)	NA	NA
Zabramski classification,^a^ type I	0.078	2.48 (0.90–6.80)	NA	NA
Lesion location, brainstem or spinal cord	**0.018**	**3.85 (1.26–11.7)**	0.268	2.62 (0.48–14.4)
Associated DVA, present	0.300	1.69 (0.63–4.56)	NA	NA
*PIK3CA* mutation, positive	**0.040**	**3.02 (1.05–8.66)**	**0.044**	**2.96 (1.03–8.49)**
*MAP3K3 *I441M, positive	0.761	0.82 (0.22–2.99)	NA	NA

^a^At the time of diagnosis *DVA,* developmental venous anomaly; *CI,* confidence interval; *NA,* not applicable

## Discussion

Surgical interventions are crucial for patients with CMs who have a high risk of hemorrhage. Screening for lesions with a high risk of bleeding among those with various characteristics is essential. Several studies have been conducted to identify the clinical and radiological characteristics of CMs associated with an elevated risk of hemorrhage [11, 12]. These studies have revealed predictive factors for hemorrhage, the most established being a history of hemorrhage and the location of the lesion [11, 12]. Previous studies have suggested that patients with mutations in CCM-associated genes exhibit specific clinical features, such as varied distribution in the CNS [1], varying numbers of lesions [7, 29], distinct MRI findings [7, 30], and different susceptibilities to hemorrhage [1, 8, 30]. However, studies on how these mutations influence the clinical behavior of CM lesions were still lacking. Thus, we conducted this study to explore the clinical and radiological characteristics of CMs with somatic *PIK3CA* and *MAP3K3* mutations, which have recently been shown to be frequently harbored by CMs [1, 5–7]. Our results showed that CMs with *PIK3CA* and *MAP3K3* mutations have some noticeable radiological tendencies. In addition, we found that the presence of the *PIK3CA* mutation is a risk factor for early (re)hemorrhage during follow-up after CM diagnosis, independent of clinical and radiological characteristics.

In the present study, *PIK3CA* mutations were identified significantly more frequently in patients with CMs associated with DVA than in those without DVA. DVAs are mostly benign, slow-flow, venous malformations composed of dilated venous channels that are often found close to CMs [33]. Although the causal relationship between CMs and DVA has not been fully clarified, a recent genetic analysis of DVAs accompanying CCM lesions with both *PIK3CA* and *MAP3K3* mutations revealed that DVAs have only *PIK3CA* mutations, indicating that the CCMs developed from DVAs that originally carried the *PIK3CA* mutation and subsequently acquired *MAP3K3 *I441M [29]. Although DVA tissues were not analyzed in the present study, the study findings also support the inference that DVA develops from vasculature that contains *PIK3CA* mutations and can be the origin of CMs.

In the present study, lesions with the *MAP3K3 *I441M mutation were observed only in patients with single lesions, and most of the lesions exhibited a Zabramski classification type II appearance. The presence of only a single lesion is characteristic of sporadic CM but not the familial type [34]. Previous studies have also indicated that this mutation is detected exclusively in sporadic cases [7, 29]. Regarding MRI appearance, Zabramski type II lesions exhibit hemorrhagic components at various times [23]. In a previous study, *MAP3K3 *I441M was correlated with Zabramski type II or III lesions [7], a finding that is consistent with the results of the present study. Type II or III lesions are characterized by a more chronic course than type I lesions, which predominantly indicates an acute/subacute hemorrhagic component [23]. This finding suggests that lesions with *MAP3K3 *I441M develop gradually rather than presenting with severe and acute bleeding. A recent study indicated that the incidence of symptomatic hemorrhage in patients with Zabramski type I lesions is significantly higher than that in patients with type II or III lesions [26]. Considering that the present study did not show a significant difference in hemorrhage between patients with *MAP3K3 *I441M and those without it or between Zabramski type I lesions and others, further research is necessary to determine the risk of hemorrhage in Zabramski type I lesions or in lesions with *MAP3K3* mutations.

The present study demonstrated that *PIK3CA* mutation is a risk factor for early (re)hemorrhage after CM diagnosis. Many studies have been conducted to identify the risk factors for hemorrhage in CMs [14, 18, 25, 35, 36]. The most established risk factors are a history of hemorrhage and lesions located in the brainstem or spinal cord [11, 16]. Although the results of the present study indicated that these factors tend to increase the risk of hemorrhage, the presence of a *PIK3CA* mutation was shown to be an additional risk factor for hemorrhage independent of those clinical factors. These findings suggest that *PIK3CA* mutation may be an inherent factor that increases the risk of hemorrhage from lesions regardless of the patient’s clinical history or lesion location in the central nervous system. A recent study showed that patients with *PIK3CA* mutations had a higher rate of hemorrhage as the initial symptom of CCM diagnosis than those with only the *MAP3K3 *I441M mutation and those with both the *PIK3CA* mutation and the *MAP3K3 *I441M [8]. However, to the best of our knowledge, this is the first study to identify somatic *PIK3CA* mutation as a risk factor for hemorrhage during follow-up for CM, independent of clinical and radiological factors. In the present study, we analyzed risk factors for hemorrhage using the mutation status in resected lesions. *PIK3CA* mutation and *MAP3K3 *I441M had no significant correlation with the size of the lesions and was observed in tiny lesions, such as those with a diameter of 5 mm. This finding indicates that *PIK3CA* and *MAP3K3* mutations likely originate in nascent CM lesions. However, it is difficult to detect the presence of somatic mutations in CMs without surgical removal. To clarify the association between somatic mutations and prognosis and to assess the indication for non-surgical treatment targeting molecular abnormalities in CMs, methods for detecting somatic mutations in CMs before surgery, such as techniques based on analysis of blood or cerebrospinal fluid samples, are needed.

This study has some limitations. First, its sample size was small. Given that this study focused on analyzing the effects of somatic mutations in CMs and the availability of mutation data depended on the quality of the tissue samples obtained, the analyses were performed by including only surgical cases in which sufficient copies of the targeted regions were available. A more extensive study conducted using higher-quality tissue specimens will yield more convincing findings. Second, this study included only surgical cases. While CM studies often involve surgical censoring, our study focused exclusively on surgical cases to analyze somatic mutations in the lesions. Therefore, the results of this study cannot be generalized to all patients with CMs. Third, mutations in CCM genes, which are well-established as being strongly associated with CMs, were not analyzed in this study. Therefore, no conclusions can be drawn regarding the effects of *PIK3CA* and *MAP3K3* mutations compared with that of CCM gene mutations. Given that *PIK3CA* and *MAP3K3* mutations may influence CM characteristics that are intricately linked to CCM gene mutations, a more detailed evaluation of their combined effects could provide a more comprehensive understanding of the genetic influences on CMs.

## Conclusions

This study demonstrated that somatic *PIK3CA* and *MAP3K3* mutations confer differences in the clinical and radiological features of patients with CMs. The results also suggest that somatic *PIK3CA* mutations may predispose CMs to (re)hemorrhage after diagnosis. These findings highlight a potential link between genetic mutations and the clinical behavior of CMs, which could inform and improve future clinical management of the disease.

## Supplementary Information

Below is the link to the electronic supplementary material. 

ESM1 (PDF 7.16 MB)

## Data Availability

No datasets were generated or analysed during the current study.
