# Supplementary material for: Clinical Significance of Somatic PIK3CA and MAP3K3 Mutations in Cerebral and Spinal Cavernous Malformations
Source: Transl Stroke Res. 2025 Jun 6;16(6):1966–74. doi: 10.1007/s12975-025-01360-2 (PMC12596314; doi:10.1007/s12975-025-01360-2)
Supplement: Supplementary file 1 — (PDF 7.16 MB) [file 12975_2025_1360_MOESM1_ESM.pdf]

Online Resources

Clinical Significance of Somatic *PIK3CA* and *MAP3K3* Mutations in Cerebral and Spinal Cavernous Malformations

Hiroki Hongo,[1] Satoru Miyawaki,[1] Keisuke Takai,[2] Hideaki Ono,[3] Masahiro Shimizu,[4] Takashi Matsukawa,[5] Shotaro Ogawa,[1] Yu Teranishi,[1] Satoshi Kiyofuji,[1] Kenta Ohara,[1] Daiichiro Ishigami,[1] Yu Sakai,[1] Seiei Torazawa,[1] Yudai Hirano,[1] Daisuke Shimada,[6] Naoto Kunii,[1,7] Seijiro Shimada,[1] Jun Mitsui,[5,8] Hiroto Katoh,[9] Daisuke Komura,[9] Hirofumi Nakatomi,[1,6] Shumpei Ishikawa,[9] Nobuhito Saito[1]

*Corresponding author:
Satoru Miyawaki, MD, PhD
Department of Neurosurgery, Faculty of Medicine, The University of Tokyo, 7-3-1 Hongo, Bunkyo-ku, Tokyo 113-8655, Japan
Tel.: +81-3-5800-8853
Fax: +81-3-5800-8655
Email: smiya-nsu@m.u-tokyo.ac.jp

This document contains the following materials:
Online Resources 1–11.

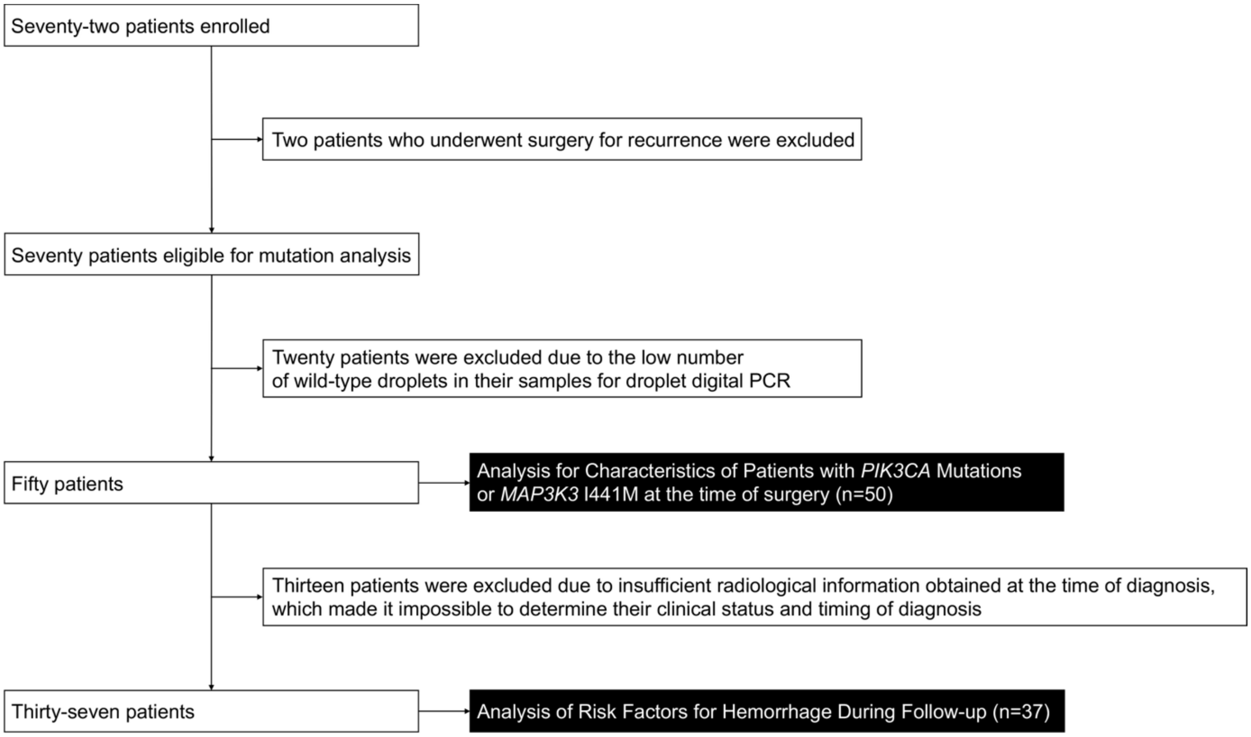

Online Resource 1 Flowchart of patient selection

In this study, we conducted two types of analyses. The first examined the characteristics of patients with *PIK3CA* mutations or the *MAP3K3* I441M variant at the time of surgery (n = 50), and the second analyzed risk factors for hemorrhage during follow-up (n = 37).

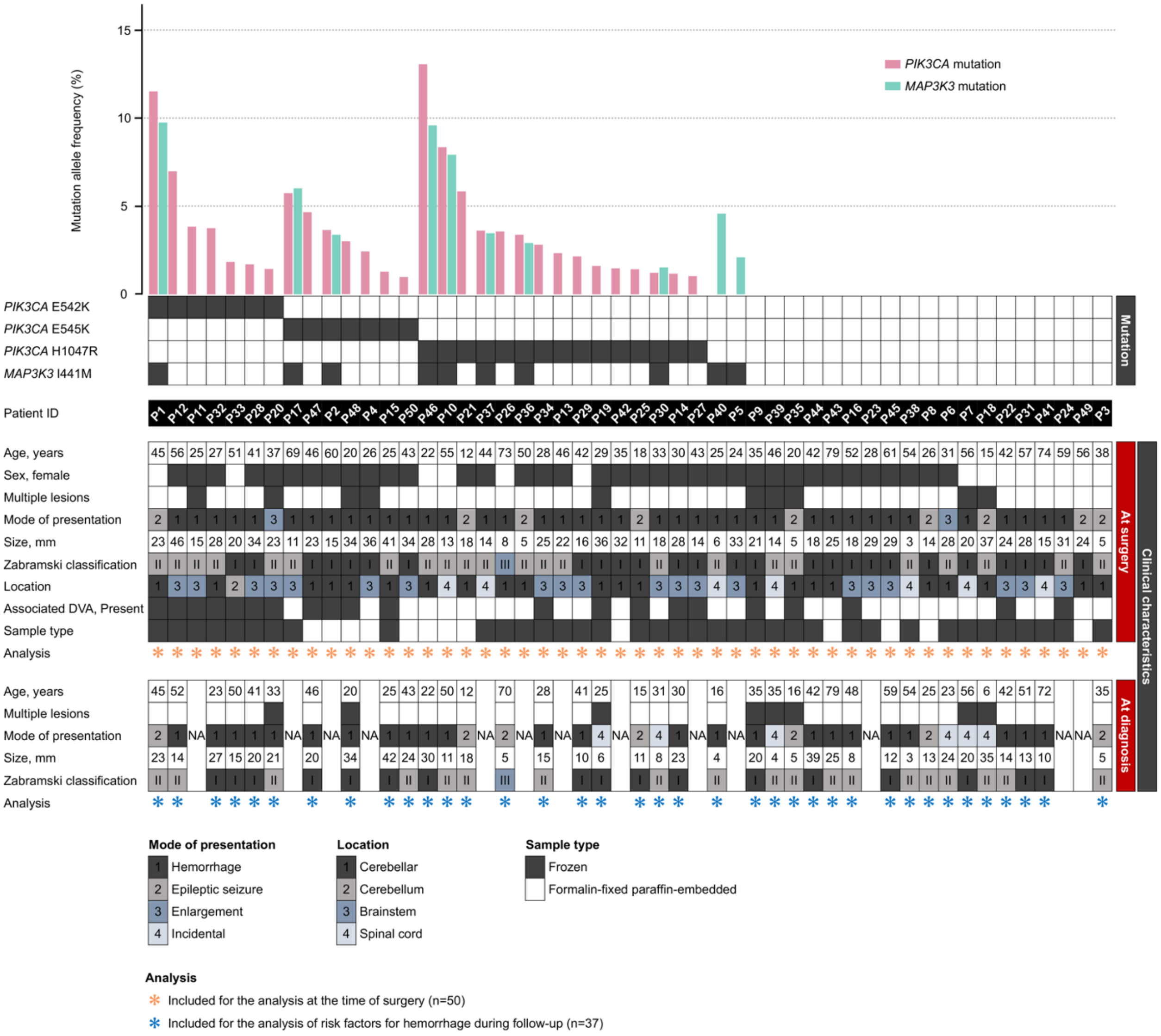

Online Resource 2 Clinical, radiological, and mutational landscapes of patients at the time of surgery and the time of diagnosis.

Patients with clinical and radiological information at the time of surgery were included in the analysis of characteristics associated with *PIK3CA* and *MAP3K3* mutations (orange asterisks, n = 50). Those with information available both at the time of surgery and at diagnosis were included in the analysis of risk factors for hemorrhage during follow-up (blue asterisks, n = 37).

DVA, developmental venous anomaly; NA, not available.

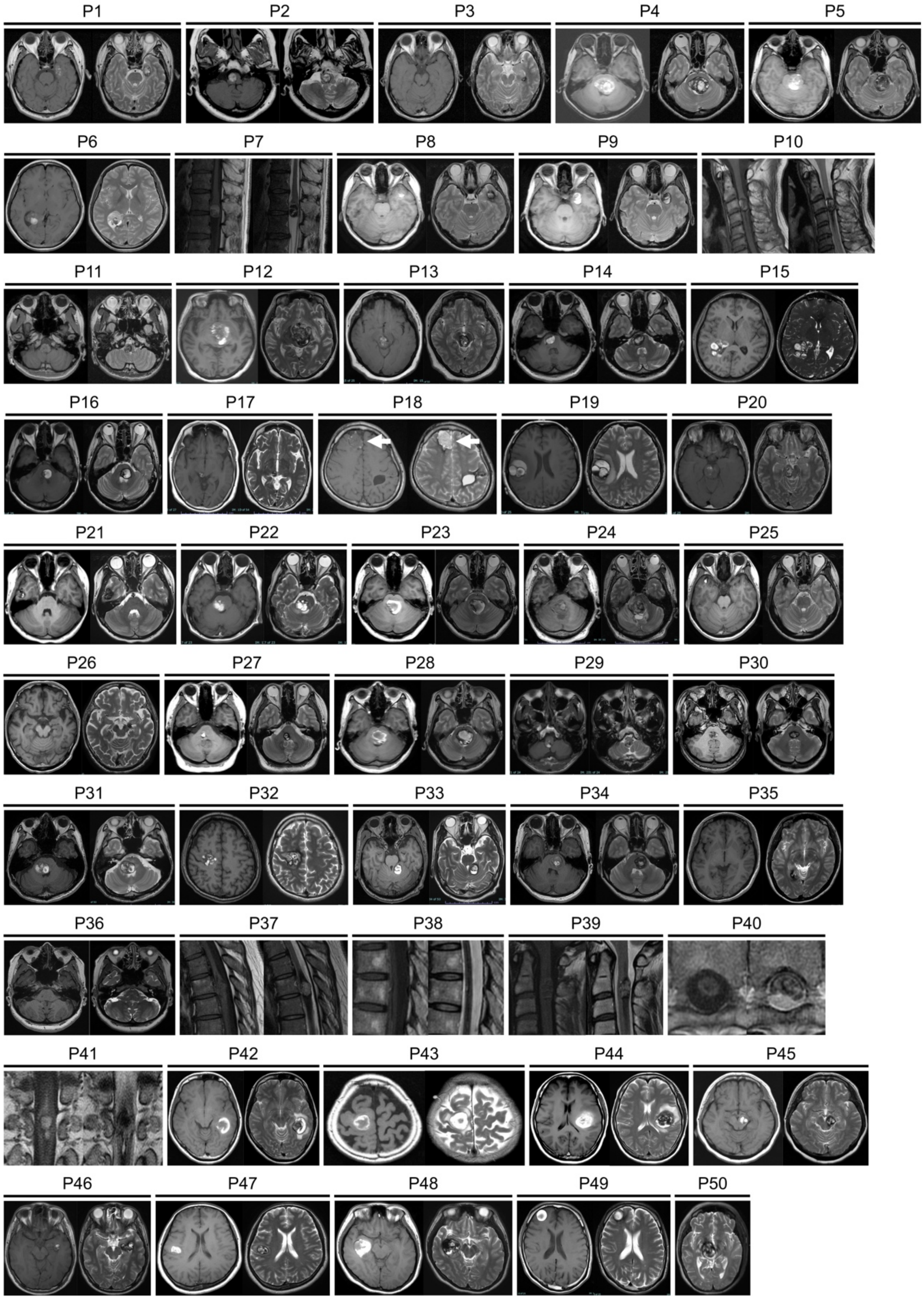

Online Resource 3 Magnetic resonance imaging findings at the time of surgery (n=50). White arrows in the image for patient 18 indicate the lesion analyzed in this study.

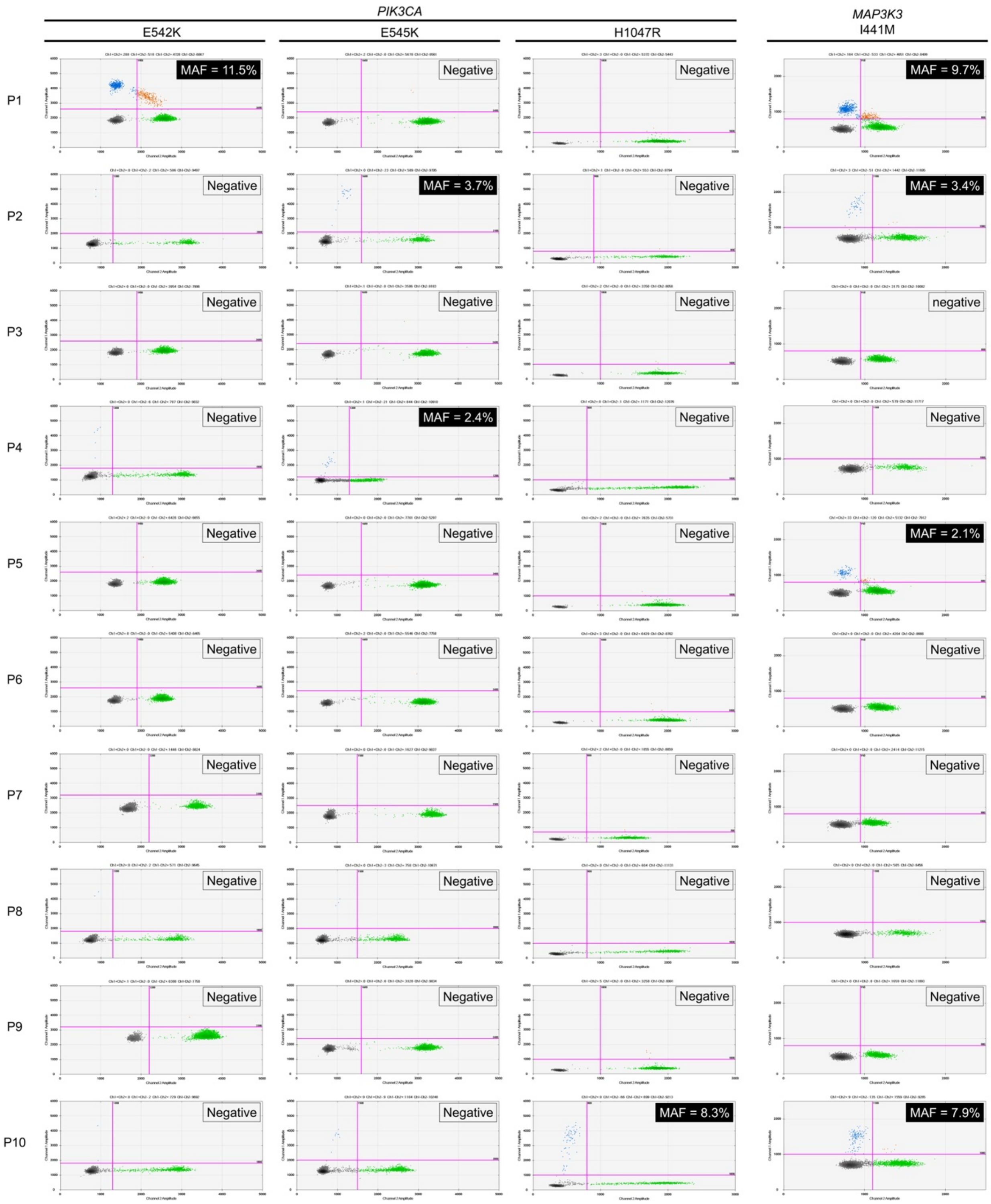
PIK3CA
E542K
E545K
H1047R
MAP3K3
I441M
P1
P2
P3
P4
P5
P6
P7
P8
P9
P10
MAF = 11.5%
MAF = 9.7%
MAF = 3.7%
MAF = 3.4%
negative
MAF = 2.4%
MAF = 2.1%
MAF = 8.3%
MAF = 7.9%
Negative

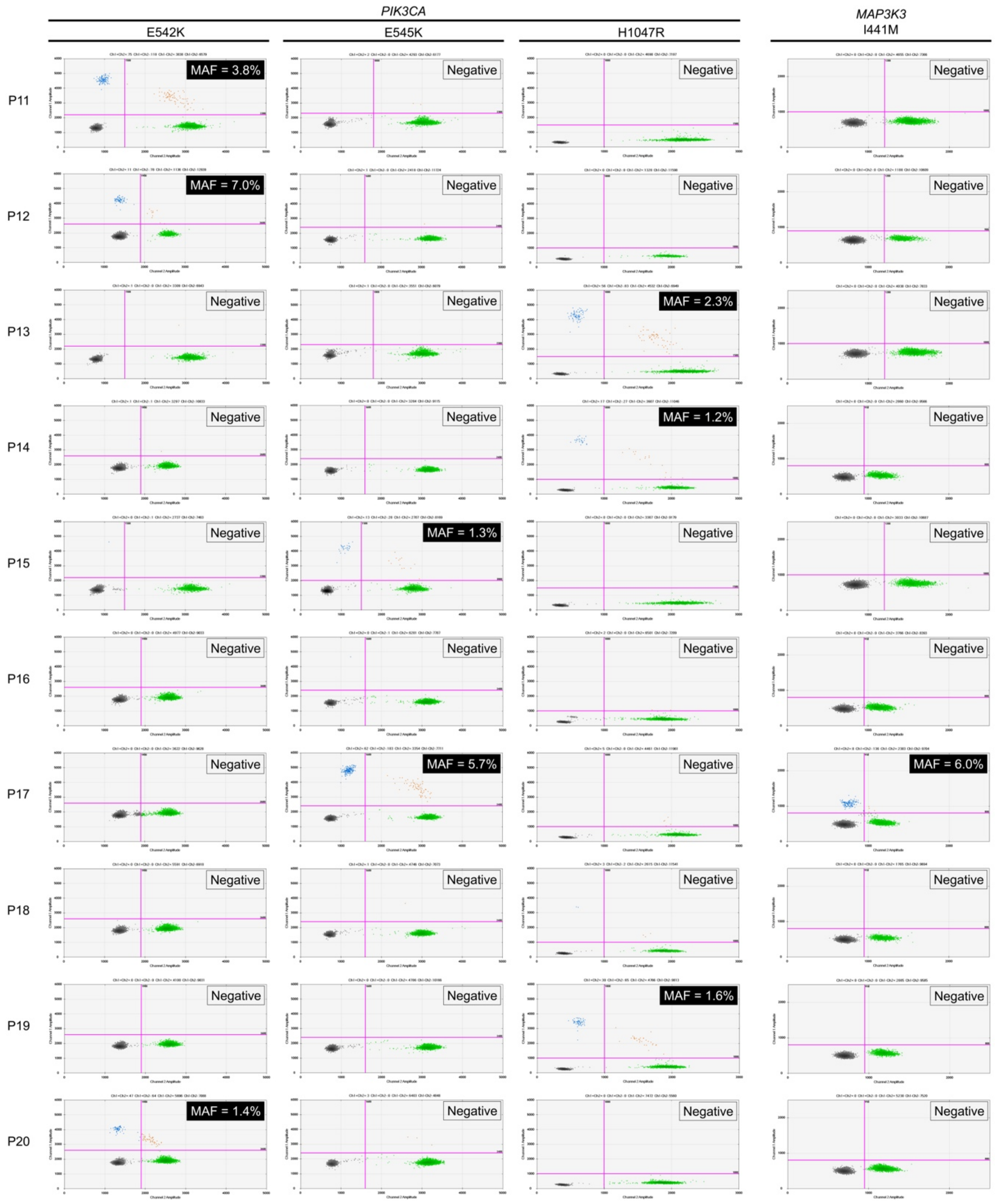
PIK3CA
E542K
E545K
H1047R
MAP3K3
I441M
P11
MAF = 3.8%
Negative
Negative
Negative
P12
MAF = 7.0%
Negative
Negative
Negative
P13
Negative
Negative
MAF = 2.3%
Negative
P14
Negative
Negative
MAF = 1.2%
Negative
P15
Negative
MAF = 1.3%
Negative
Negative
P16
Negative
Negative
Negative
Negative
P17
Negative
MAF = 5.7%
Negative
MAF = 6.0%
P18
Negative
Negative
Negative
Negative
P19
Negative
Negative
MAF = 1.6%
Negative
P20
MAF = 1.4%
Negative
Negative
Negative

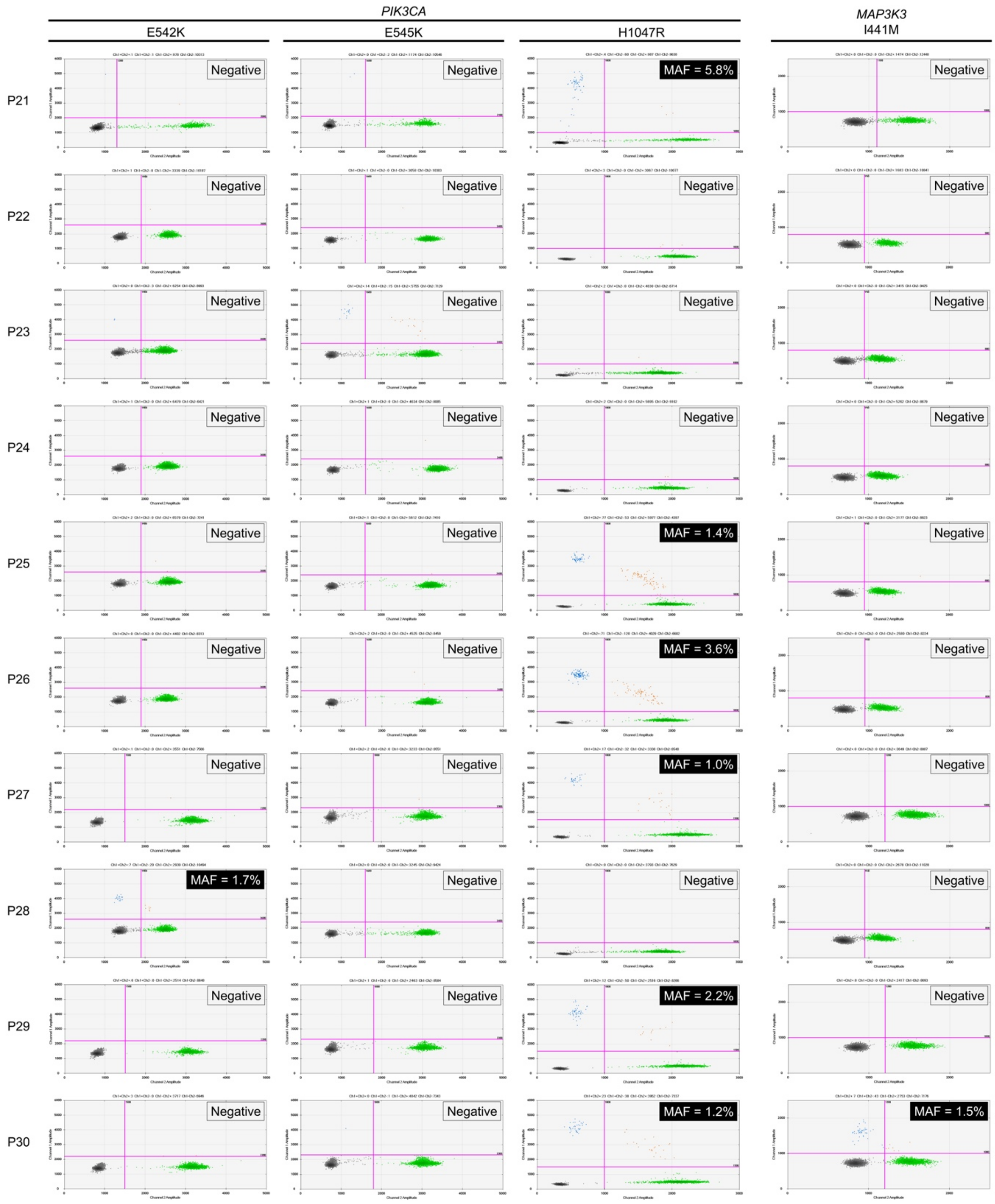
PIK3CA
MAP3K3
E542K
E545K
H1047R
I441M
P21
Negative
Negative
MAF = 5.8%
Negative
P22
Negative
Negative
Negative
Negative
P23
Negative
Negative
Negative
Negative
P24
Negative
Negative
Negative
Negative
P25
Negative
Negative
MAF = 1.4%
Negative
P26
Negative
Negative
MAF = 3.6%
Negative
P27
Negative
Negative
MAF = 1.0%
Negative
P28
MAF = 1.7%
Negative
Negative
Negative
P29
Negative
Negative
MAF = 2.2%
Negative
P30
Negative
Negative
MAF = 1.2%
MAF = 1.5%

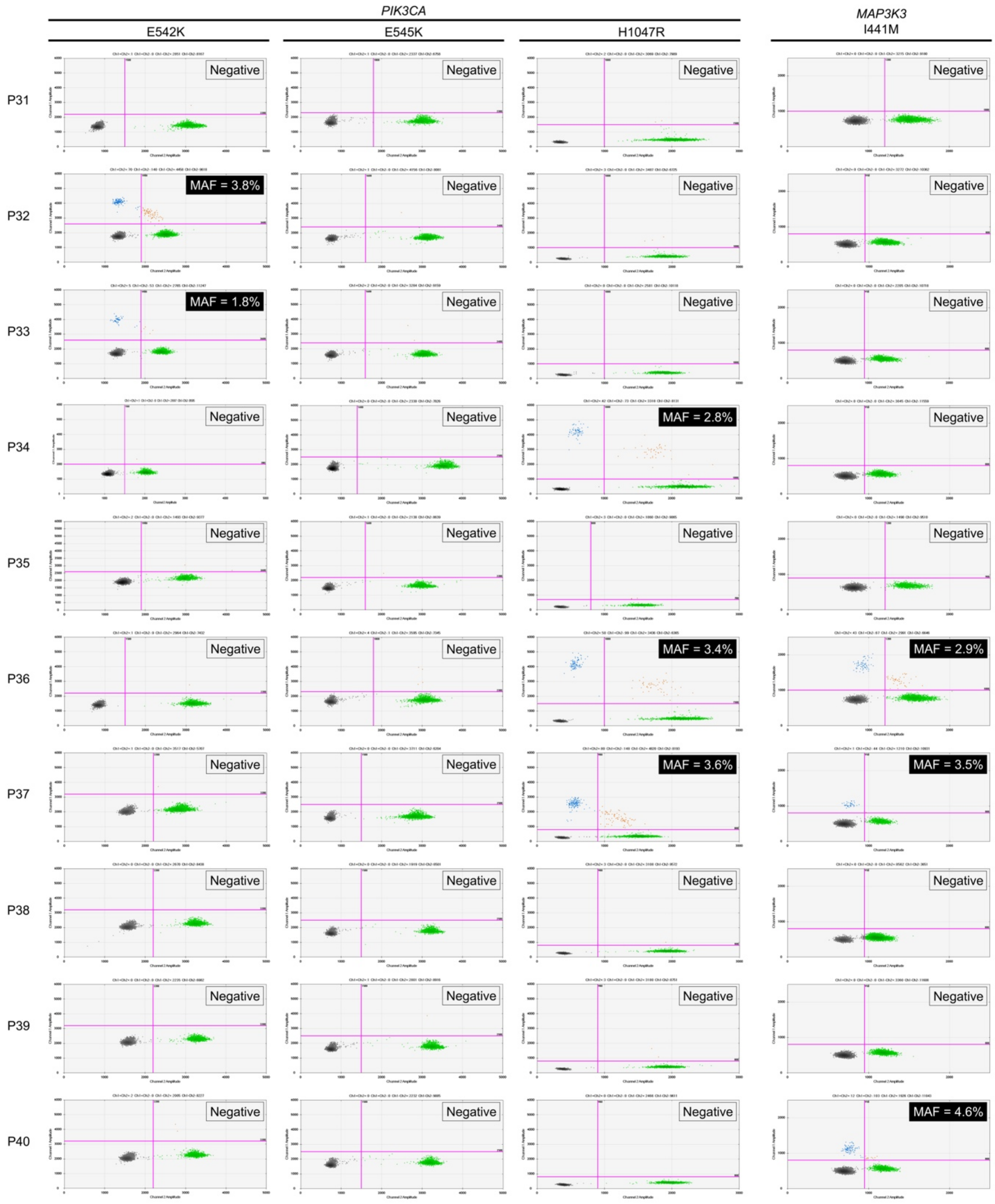
PIK3CA
MAP3K3
E542K
E545K
H1047R
I441M
P31
P32
P33
P34
P35
P36
P37
P38
P39
P40
Negative
MAF = 3.8%
MAF = 1.8%
MAF = 2.8%
MAF = 3.4%
MAF = 2.9%
MAF = 3.6%
MAF = 3.5%
MAF = 4.6%

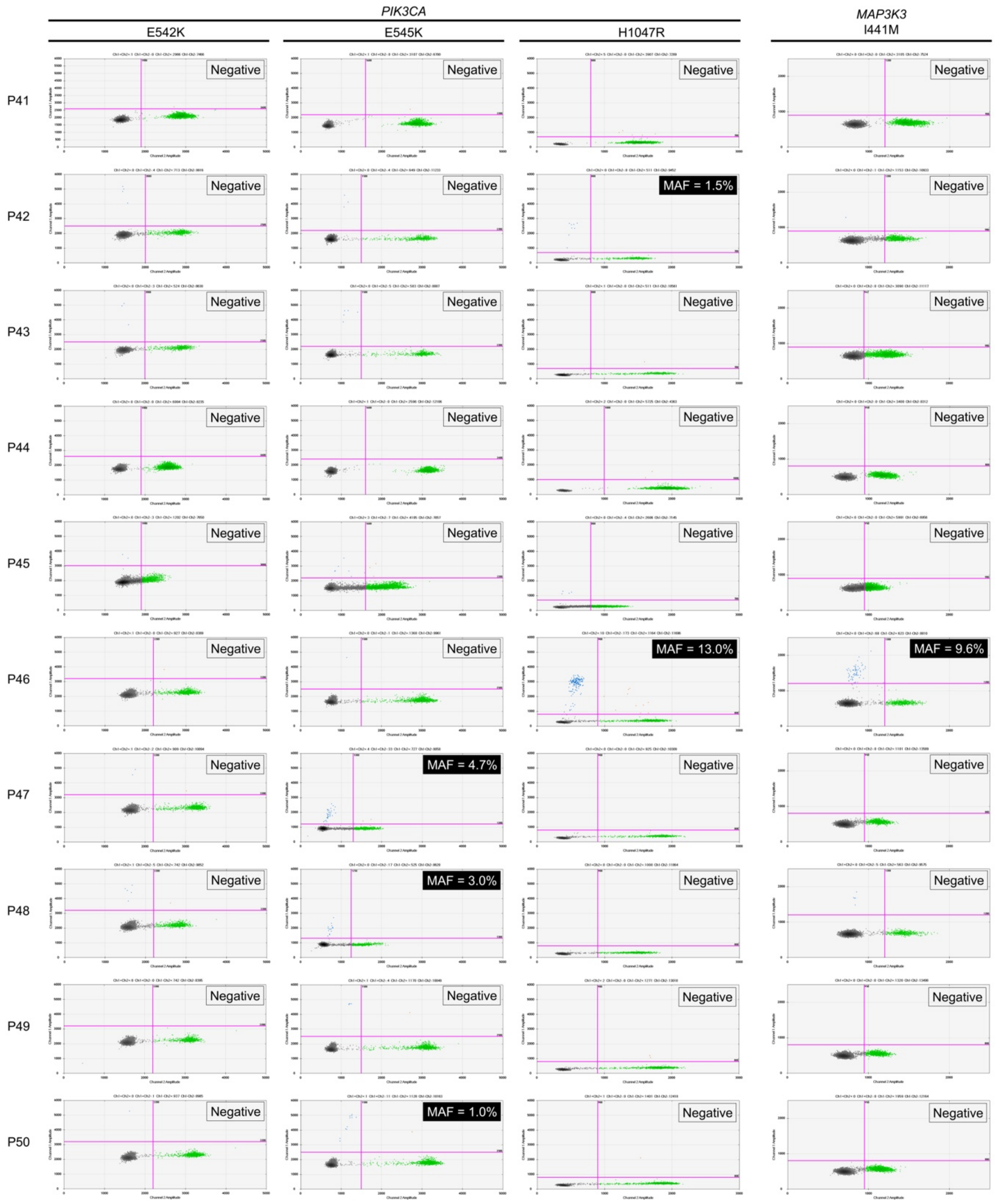

Online Resource 4 Scatter plots of ddPCRs for *PIK3CA* and *MAP3K3* mutations (n=50). Each dot represents a droplet, with blue indicating mutation positive, green indicating wild-type positive, orange indicating positive for both, and black indicating negative for both. Boxed numbers represent the fractional abundance of mutation alleles in each sample.

ddPCR, droplet digital polymerase chain reaction; MAF, mutation allele frequency.

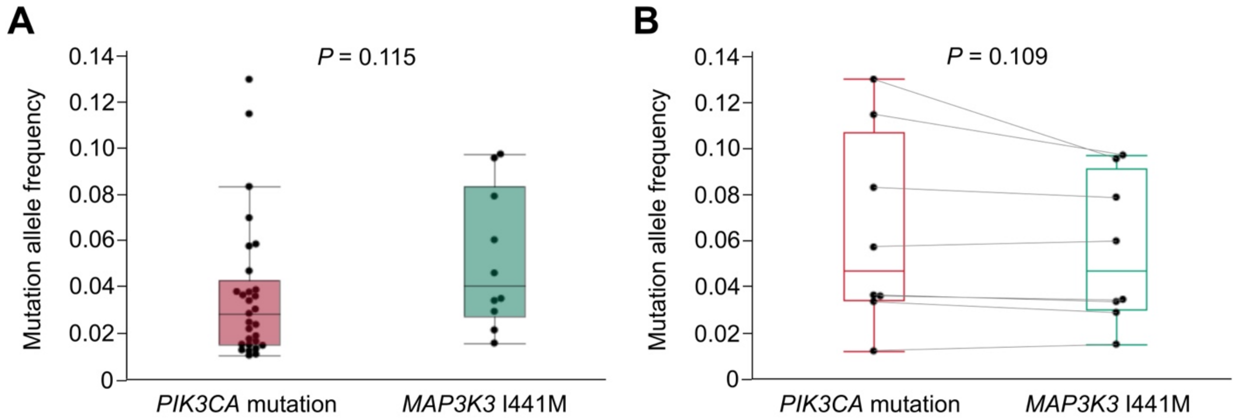

Online Resource 5 Comparison of mutation allele frequencies (MAFs) between *PIK3CA* and *MAP3K3* mutations. (A) Comparison of MAFs between *PIK3CA* mutations and *MAP3K3* I441M among all mutations identified. (B) Comparison of MAFs between *PIK3CA* mutations and *MAP3K3* I441M among mutations identified in patients with both *PIK3CA* and *MAP3K3* mutations. The lines connect *PIK3CA* mutations and *MAP3K3* I441M in a single sample.

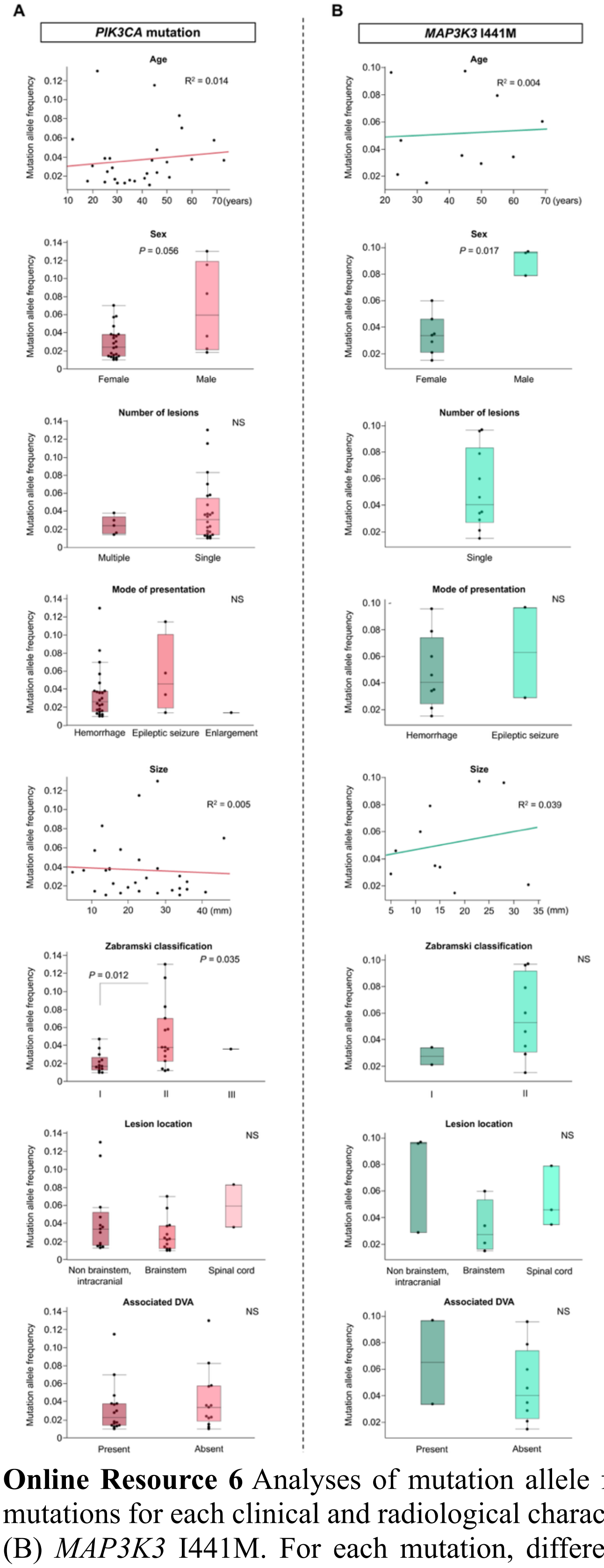

Online Resource 6 Analyses of mutation allele frequencies (MAFs) of *PIK3CA* and *MAP3K3* mutations for each clinical and radiological characteristic. Analysis of (A) *PIK3CA* mutations and (B) *MAP3K3* I441M. For each mutation, differences in MAFs were compared across various clinical and radiological characteristics. NS, not significant.

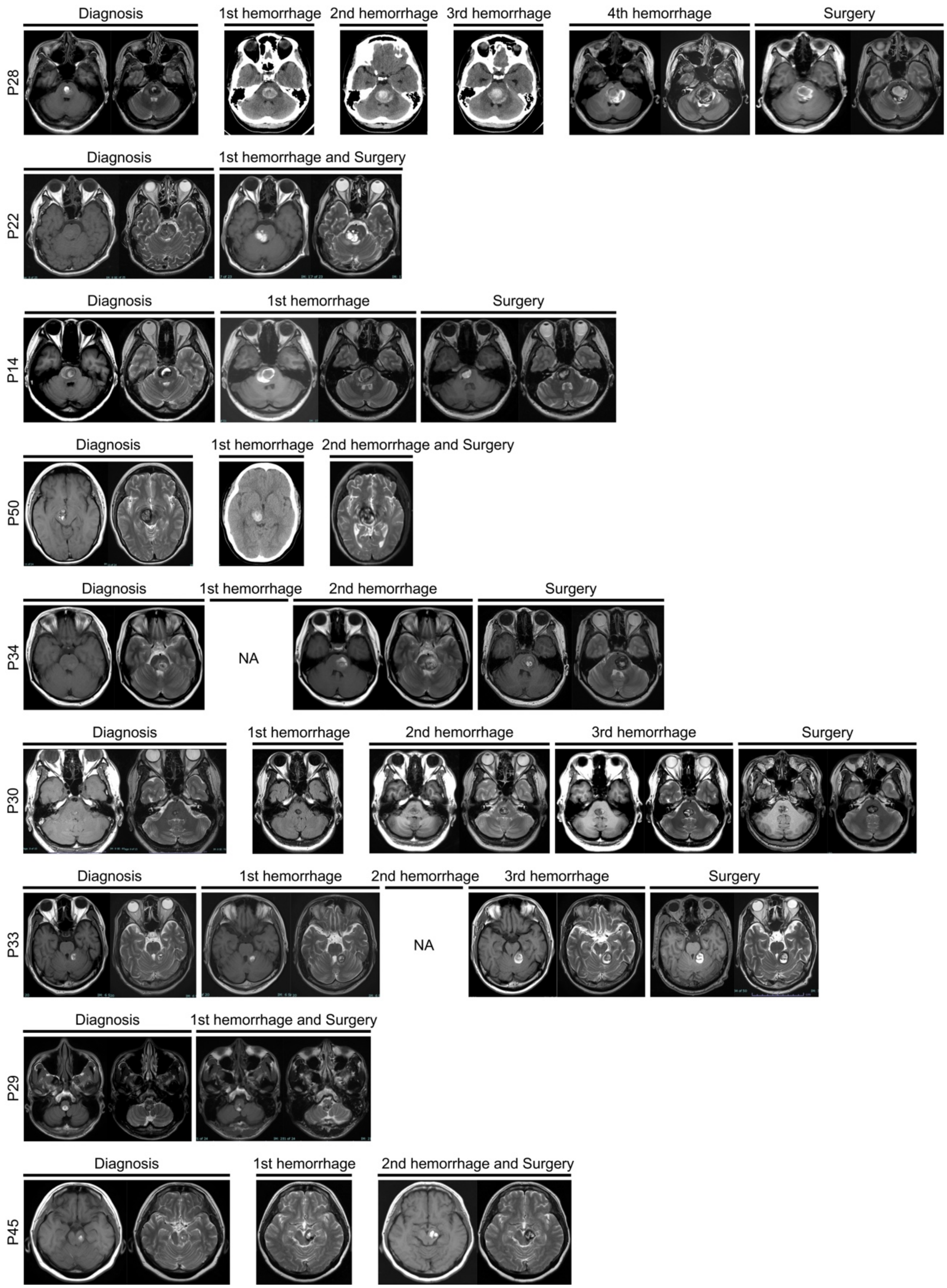
Diagnosis
1st hemorrhage
2nd hemorrhage
3rd hemorrhage
4th hemorrhage
Surgery
P28
Diagnosis
1st hemorrhage and Surgery
P22
Diagnosis
1st hemorrhage
Surgery
P14
Diagnosis
1st hemorrhage
2nd hemorrhage and Surgery
P50
Diagnosis
1st hemorrhage
2nd hemorrhage
Surgery
NA
P34
Diagnosis
1st hemorrhage
2nd hemorrhage
3rd hemorrhage
Surgery
P30
Diagnosis
1st hemorrhage
2nd hemorrhage
3rd hemorrhage
Surgery
NA
P33
Diagnosis
1st hemorrhage and Surgery
P29
Diagnosis
1st hemorrhage
2nd hemorrhage and Surgery
P45

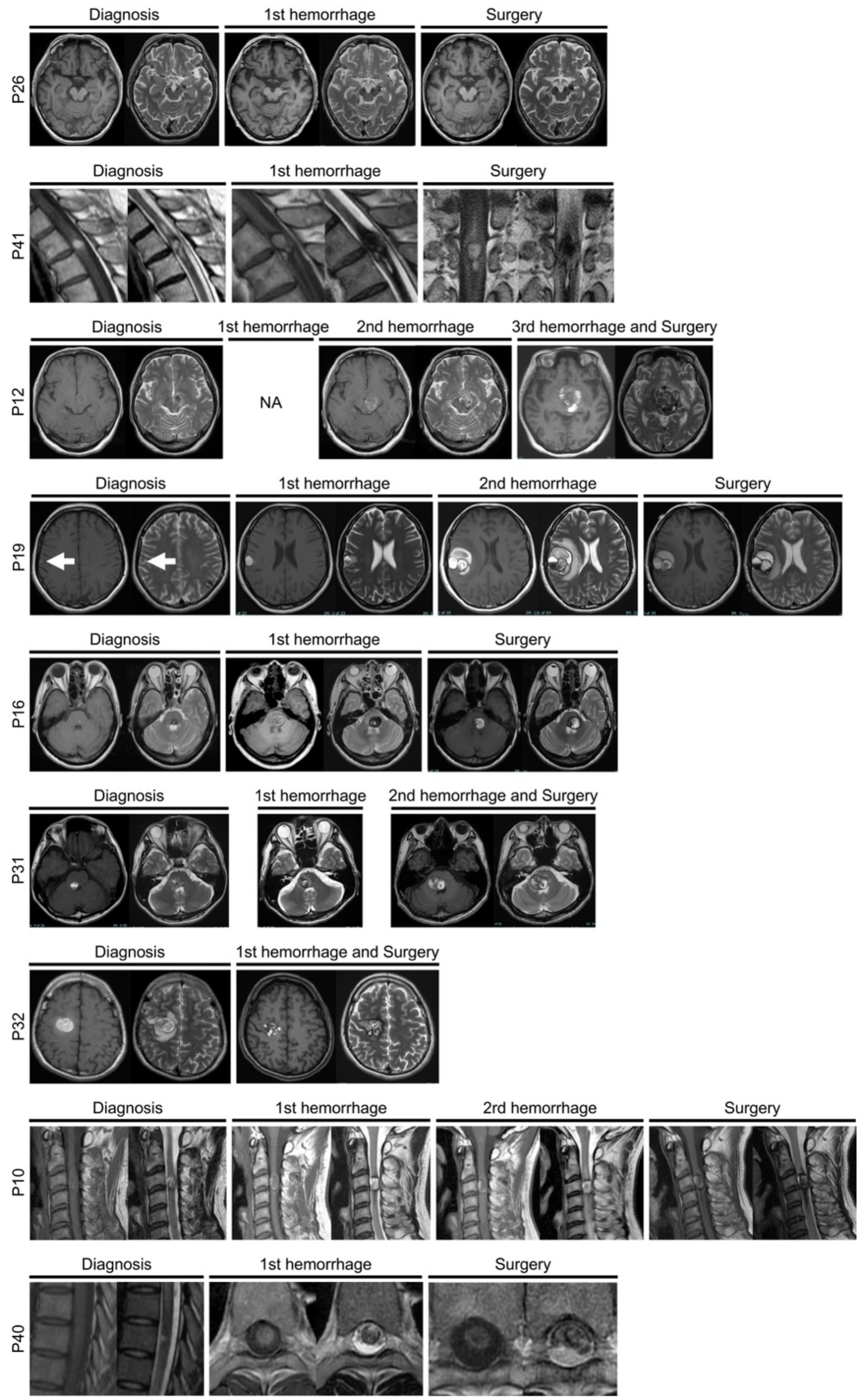

Online Resource 7 MRI and CT images from 18 of the 37 patients who were included in the analysis of risk factors for hemorrhage during follow-up and experienced hemorrhage after diagnosis. Patients are arranged in order of time to hemorrhage, from shortest to longest duration after diagnosis. White arrows in the image for patient 19 indicate the lesion analyzed in this study. NA, not available; MRI, magnetic resonance imaging; CT, computed tomography.

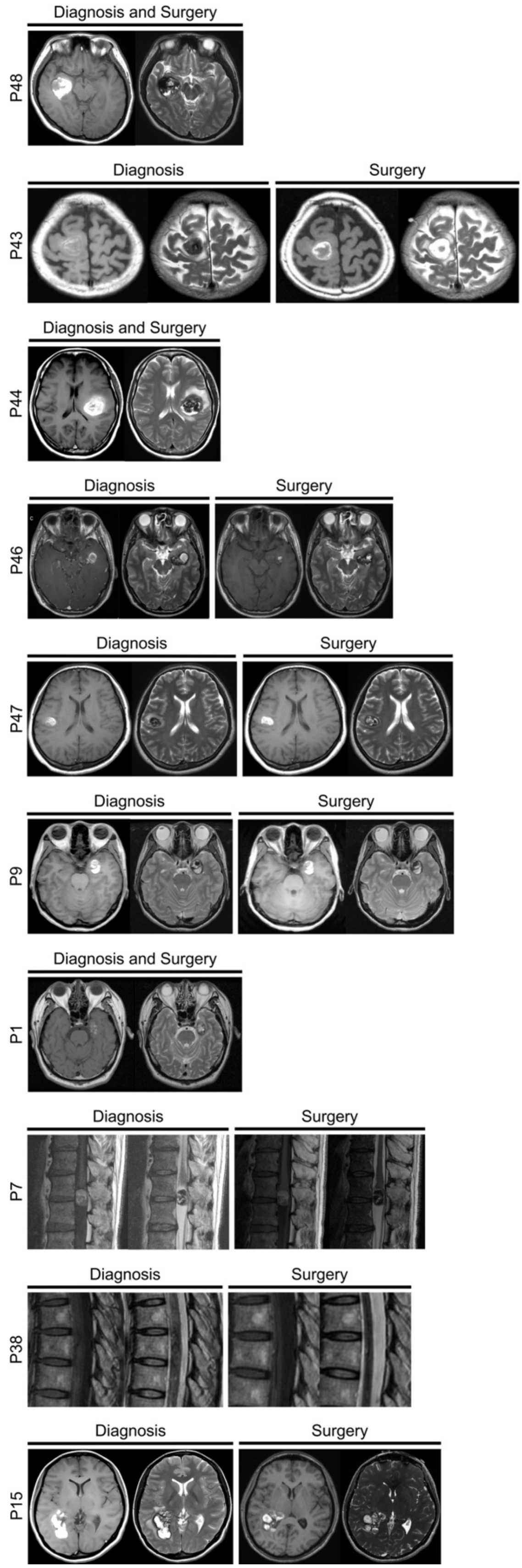
Diagnosis and Surgery
P48
Diagnosis
Surgery
P43
Diagnosis and Surgery
P44
Diagnosis
Surgery
P46
Diagnosis
Surgery
P47
Diagnosis
Surgery
P9
Diagnosis and Surgery
P1
Diagnosis
Surgery
P7
Diagnosis
Surgery
P38
Diagnosis
Surgery
P15

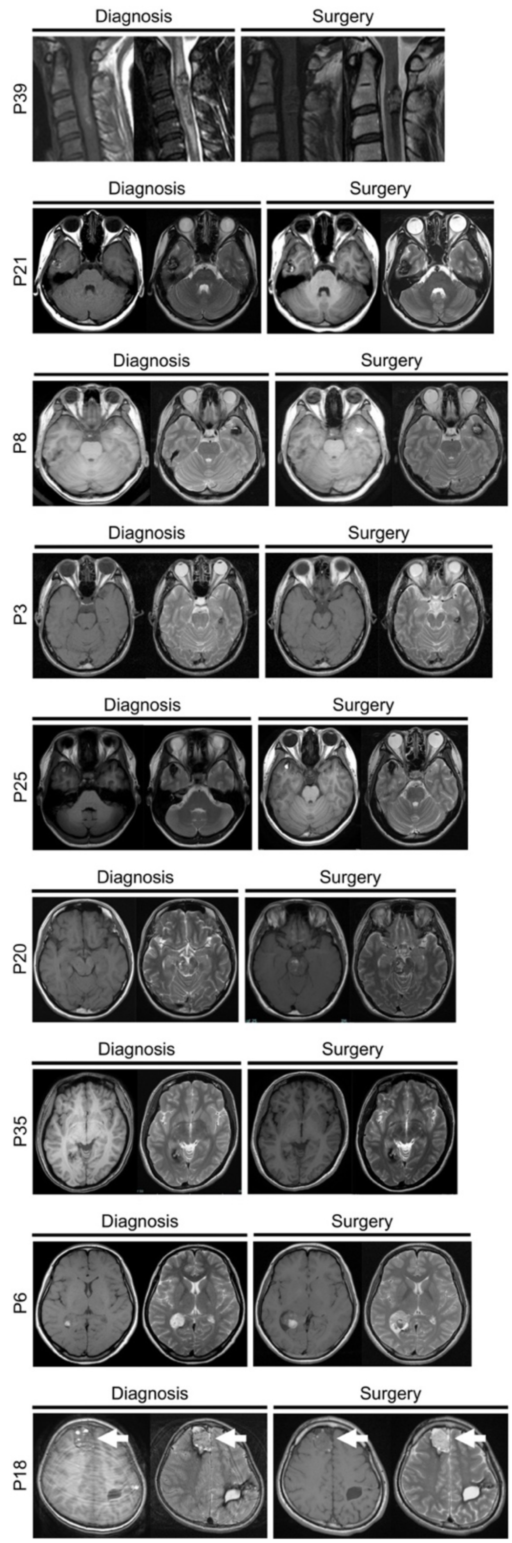

Online Resource 8 MRI and CT images from 19 of the 37 patients who were included in the analysis of risk factors for hemorrhage during follow-up and did not experience hemorrhage after diagnosis. Patients are arranged in order of time to surgery, from shortest to longest duration after diagnosis. White arrows in the image for patient 18 indicate the lesion analyzed in this study. MRI, magnetic resonance imaging.

Patient ID	At diagnosis								At surgery	Mutation	
	Age	Sex	Number of lesions	Mode of presentation	Lesion size	Zabramski classification	Lesion location	Associated DVA	Mode of presentation	*PIK3CA* mutation	*MAP3K3* I441M
28	41	Female	Single	Hemorrhage	20	I	Brainstem	Present	Hemorrhage	Positive	Negative
22	42	Male	Single	Hemorrhage	14	II	Brainstem	Present	Hemorrhage	Negative	Negative
14	30	Female	Single	Hemorrhage	23	I	Brainstem	Present	Hemorrhage	Positive	Negative
50	43	Female	Single	Hemorrhage	24	II	Brainstem	Absent	Hemorrhage	Positive	Negative
34	28	Female	Single	Hemorrhage	15	II	Brainstem	Present	Hemorrhage	Positive	Negative
30	31	Female	Single	Incidental	8	II	Brainstem	Absent	Hemorrhage	Positive	Positive
33	50	Male	Single	Hemorrhage	15	I	Cerebellum	Present	Hemorrhage	Positive	Negative
29	41	Male	Single	Hemorrhage	10	I	Brainstem	Absent	Hemorrhage	Positive	Negative
45	59	Female	Single	Hemorrhage	12	I	Brainstem	Absent	Hemorrhage	Negative	Negative
26	70	Male	Single	Epileptic seizure	5	III	Cerebellar	Absent	Hemorrhage	Positive	Negative
41	72	Male	Single	Hemorrhage	10	I	Spinal cord	Absent	Hemorrhage	Negative	Negative
12	52	Female	Single	Hemorrhage	14	II	Brainstem	Present	Hemorrhage	Positive	Negative
19	25	Female	Multiple	Incidental	6	I	Cerebellar	Present	Hemorrhage	Positive	Negative
16	48	Female	Single	Hemorrhage	8	II	Brainstem	Present	Hemorrhage	Negative	Negative
31	51	Male	Single	Hemorrhage	13	I	Brainstem	Absent	Hemorrhage	Negative	Negative
32	23	Female	Single	Hemorrhage	27	I	Cerebellar	Present	Hemorrhage	Positive	Negative
10	50	Male	Single	Hemorrhage	11	II	Spinal cord	Absent	Hemorrhage	Positive	Positive
40	16	Female	Single	Hemorrhage	4	II	Spinal cord	Absent	Hemorrhage	Negative	Positive
48	20	Female	Multiple	Hemorrhage	34	I	Cerebellar	Present	Hemorrhage*	Positive	Negative
43	79	Female	Single	Hemorrhage	25	II	Cerebellar	Absent	Hemorrhage*	Negative	Negative
44	42	Female	Single	Hemorrhage	39	I	Cerebellar	Absent	Hemorrhage*	Negative	Negative
46	22	Male	Single	Hemorrhage	30	I	Cerebellar	Absent	Hemorrhage*	Negative	Positive
47	46	Female	Single	Hemorrhage	20	I	Cerebellar	Present	Hemorrhage*	Positive	Negative
9	35	Female	Multiple	Hemorrhage	20	I	Cerebellar	Present	Epileptic seizure	Negative	Negative
1	45	Male	Single	Epileptic seizure	23	II	Cerebellar	Present	Epileptic seizure	Positive	Positive
7	56	Male	Multiple	Hemorrhage	20	I	Spinal cord	Absent	Hemorrhage*	Negative	Negative
38	54	Female	Single	Hemorrhage	3	II	Spinal cord	Absent	Hemorrhage*	Negative	Negative
15	25	Female	Single	Hemorrhage	42	I	Cerebellar	Present	Hemorrhage*	Positive	Negative
39	35	Female	Multiple	Hemorrhage	4	II	Spinal cord	Absent	Hemorrhage*	Negative	Negative
21	12	Female	Single	Epileptic seizure	18	II	Cerebellar	Absent	Epileptic seizure	Positive	Negative
8	25	Female	Single	Epileptic seizure	13	II	Cerebellar	Absent	Epileptic seizure	Negative	Negative
3	35	Male	Single	Epileptic seizure	5	II	Cerebellar	Absent	Epileptic seizure	Negative	Negative
25	15	Female	Single	Epileptic seizure	11	I	Cerebellar	Present	Epileptic seizure	Positive	Negative
20	33	Female	Multiple	Hemorrhage	21	II	Brainstem	Present	Enlargement	Positive	Negative
35	16	Female	Multiple	Epileptic seizure	5	II	Cerebellar	Present	Epileptic seizure	Negative	Negative
6	23	Female	Single	Incidental	24	II	Cerebellar	Absent	Enlargement	Negative	Negative
18	6	Male	Multiple	Incidental	35	II	Cerebellar	Absent	Epileptic seizure	Negative	Negative

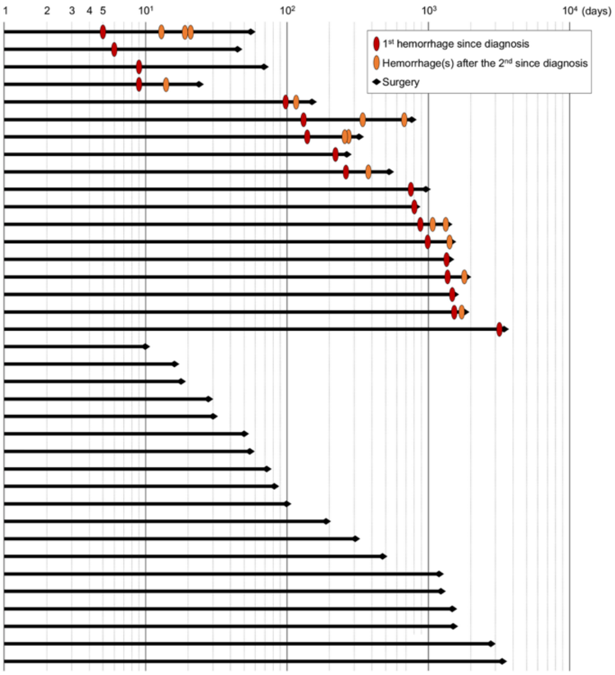

*Identical to hemorrhage at diagnosis
DVA indicates developmental venous anomaly

Online Resource 9 Clinical course of patients from the time of diagnosis (n=37)

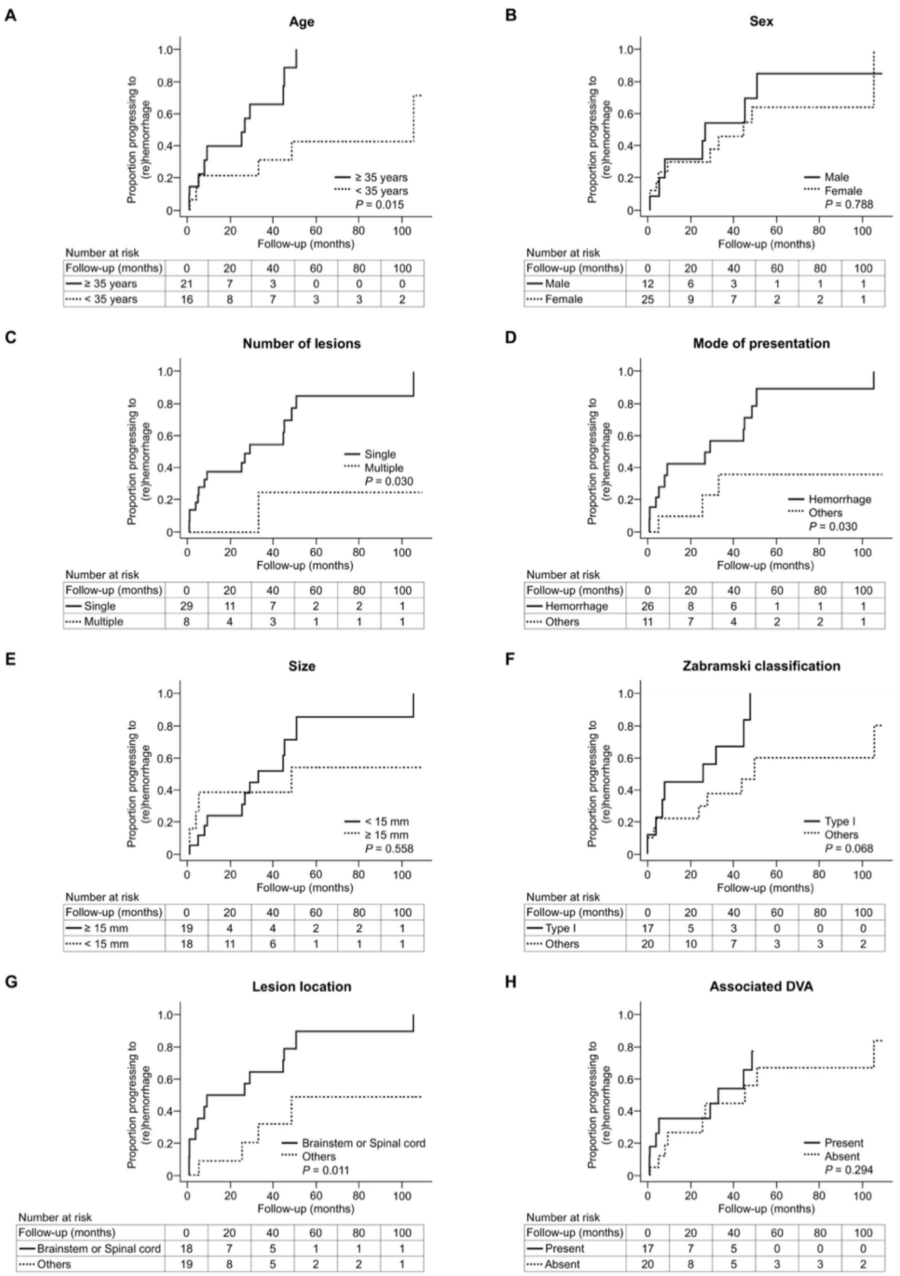

Online Resource 10 Kaplan–Meier plots of progression to intracerebral/spinal (re)hemorrhage in the analysis of risk factors for hemorrhage during follow-up (n=37). Plots of patients stratified according to (A) age, (B) sex, (C) number of lesions, (D) mode of presentation, (E) lesion size, (F) Zabramski classification, (G) lesion location, and (H) associated DVA. The horizontal axis represents follow-up time (months), and the vertical axis represents the proportion of patients progressing to hemorrhage after diagnosis. These plots illustrate the cumulative hemorrhage rate (1 − survival rate) over time. The "number at risk" at each time point indicates the number of individuals who have not yet experienced the event up to that point. In panels A, C, D, and G, the differences first reached statistical significance at 46 months (P = 0.027), 51 months (P = 0.048), 51 months (P = 0.046), and 1 month (P = 0.032), respectively, and all remained significant thereafter.
DVA, developmental venous anomaly.

Online Resource 11 Time Points at Which the Differences Become Statistically Significant in Kaplan–Meier Analyses in the Analysis of Risk Factors for Hemorrhage During Follow-up (n=37)

**Follow-up (months)**	***p* value**									
	***PIK3CA* mutation**[a]	***MAP3K3* I441M**[a]	**Age**[b]	**Sex**[c]	**Number of lesions**[d]	**Mode of presenta-tion**[e]	**Size**[f]	**Zabramski classifica-tion**[g]	**Lesion location**[h]	**Associated DVA**[i]
**1**									0.032	
**10**	0.091	0.893	0.350	0.963	0.107	0.082	0.281	0.352	0.012	0.368
**20**	0.091	0.893	0.350	0.963	0.107	0.082	0.281	0.352	0.012	0.368
**30**	0.066	0.628	0.07	0.596	0.051	0.088	0.681	0.415	0.013	0.632
**33**	0.037									
**40**	0.037	0.550	0.119	0.753	0.155	0.178	0.844	0.243	0.030	0.459
**45**	0.065									
**46**			0.027							
**50**	0.068	0.299	0.035	0.774	0.058	0.072	0.923	0.068	0.027	0.294
**51**	0.031				0.048	0.046				
**60**	0.031	0.515	0.015	0.594	0.048	0.046	0.727	0.068	0.017	0.294
**70**	0.031	0.515	0.015	0.594	0.048	0.046	0.727	0.068	0.017	0.294
**80**	0.031	0.515	0.015	0.594	0.048	0.046	0.727	0.068	0.017	0.294
**90**	0.031	0.515	0.015	0.594	0.048	0.046	0.727	0.068	0.017	0.294
**100**	0.031	0.515	0.015	0.594	0.048	0.046	0.727	0.068	0.017	0.294
**110**	0.031	0.760	0.015	0.788	0.030	0.030	0.558	0.068	0.011	0.294

[a]Comparison between positive and negative; [b]Comparison between <35 years and ≥35 years; [c]Comparison between female and male; [d]Comparison between single and multiple; [e]Comparison between hemorrhage and others; [f]Comparison between <15 mm and ≥15 mm; [g]Comparison between type I and others; [h]Comparison between brainstem/spinal cord and others; [I]Comparison between present and absent

P values were calculated using the log-rank test. Follow-up months shown in blue cells indicate the time points at which the p value dropped below or rose above 0.05 for a given factor. The orange cells represent those with p-values below 0.05. Abbreviation: DVA, developmental venous anomaly
